# Epidemiological trends for functional pancreatic neuroendocrine tumors: A study combining multiple imputation with age adjustment

**DOI:** 10.3389/fendo.2023.1123642

**Published:** 2023-04-11

**Authors:** Shuaiwu Luo, Jiakun Wang, Linquan Wu, Cong Wang, Jun Yang, Min Li, Ligan Zhang, Jin Ge, Chi Sun, Enliang Li, Jun Lei, Yuting Liao, Fan Zhou, Wenjun Liao

**Affiliations:** ^1^ Department of Hepatological and Pancreatic Surgery, The Second Affiliated Hospital of Nanchang University, Nanchang, China; ^2^ Department of General Surgery, The Second Affiliated Hospital of Nanchang University, Nanchang, China; ^3^ Department of Nursing, Gannan Medical College, Ganzhou, China

**Keywords:** functional pancreatic neuroendocrine tumors, epidemiology, incidence, mortality, SEER

## Abstract

**Purpose:**

The purpose of this study was to examine trends in the incidence and incidence-based (IB) mortality of functional pancreatic neuroendocrine tumors(F-PNETs), and to identify factors associated with survival times.

**Methods:**

Data were obtained from the Surveillance, Epidemiology, and End Results database from 2000 to 2017. Trends in the age-adjusted incidence of F-PNETs and IB mortality were examined using the Joinpoint Regression Program. Statistical analyses were run using chi-square tests, Kaplan–Meier curves, and the Cox proportional hazards model. Multiple imputation was used to deal with missing data.

**Results:**

A total of 142 patients with F-PNETs met the study inclusion criteria. It was found that the incidence of F-PNETs decreased over the study period, with an annual percent change (APC) of -2. 5% (95% CI [-4. 3, -0. 5], P<0. 05). This decrease was found to be significant for women, and also when limited to cases with distant disease or rare F-PNETs, with APCs of -4. 2% (95% CI [-7. 4, -0. 9], P<0. 05), -6. 7% (95% CI [-10. 4, -2. 8], P<0. 05), and -9. 1% (95% CI [-13. 5, -4. 4], P<0. 05), respectively. The Cox regression analysis revealed that the tumor size, tumor stage, tumor type, and surgical resection were associated with F-PNETs mortality.

**Conclusions:**

This was the first population-based epidemiological study of F-PNETs and we found a continual decrease in the incidence of F-PNETs from 2000 to 2017. The prognosis and survival times were closely related to the calendar year at diagnosis, tumor stage, and tumor size.

## Introduction

Pancreatic neuroendocrine tumors (PNETs) are a heterogeneous group of tumors that arise from the neurons and endocrine cells of the pancreas. Although PNETs are rare tumors, the incidence of the disease is currently increasing (1). PNETs can be either functional (F-PNETs), where they secrete hormones, or non-functional (NF-PNETs), where they do not secrete hormones (2). F-PNETs are relatively rare, accounting for approximately 20% of all PNETs (3). Insulinomas and gastrinomas are the most usual F-PNETs; rare types include glucagonomas, VIPomas, and somatostatinomas (rare F-PNETs; RF-PNETs). In older studies insulinomas and NF-PNETs were approximately equally frequent making up 1/3 of all PNETs each, with gastrinomas slightly less frequent (4). However, Recently NF-PNETs are being increasing reported and these are making up twice the above percentages of PNETs in some recent studies (4, 5). This is a marked change from the past. Therefore, more studies tend to focus on NF-PNETs, resulting in less attention to F-PNETs in recent years.

The diagnosis of F-PNETs requires clinical symptoms of hormone overexpression in addition to abnormal immunohistochemical results (2). However, misdiagnosis is common because the symptoms overlap with other diseases. Surgery is the standard treatment for F-PNETs, and the prognosis is good. However, if the disease is locally advanced or has metastasized, there is significant mortality and the treatment options have limited efficacy (6). It is therefore of utmost importance to ensure early diagnosis. This can be facilitated by recognizing the underlying risk factors and understanding the disease pathogenesis. As epidemiological data can reveal important information that is of relevance, it is timely to conduct a detailed study.

Little is currently known about the epidemiological characteristics, incidence, and mortality of F-PNETs, presumably because of the multiple subtypes and complex classification system. There is therefore a lack of epidemiological data of relevance for clinical diagnosis and treatment. Some studies have shown that the overall incidence of PNETs or NF-PNETs is increasing (7, 8). However, few epidemiological studies have considered F-PNETs separately, so the incidence, trends, and mortality are currently unclear. There is also a lack of data concerning RF-PNETs.

In this study, we examined the epidemiological trends for F-PNETs and identified prognostic factors in the first time. The study used data from the Surveillance, Epidemiology, and End Results (SEER) database with multiple imputation, and annual percent changes (APCs) were calculated to quantify the changes over time. The study is of relevance for understanding of the pathogenesis of F-PNETs and could be of use in clinical practice and for developing screening guidelines.

## Methods

### Data source

**Figure 1:**
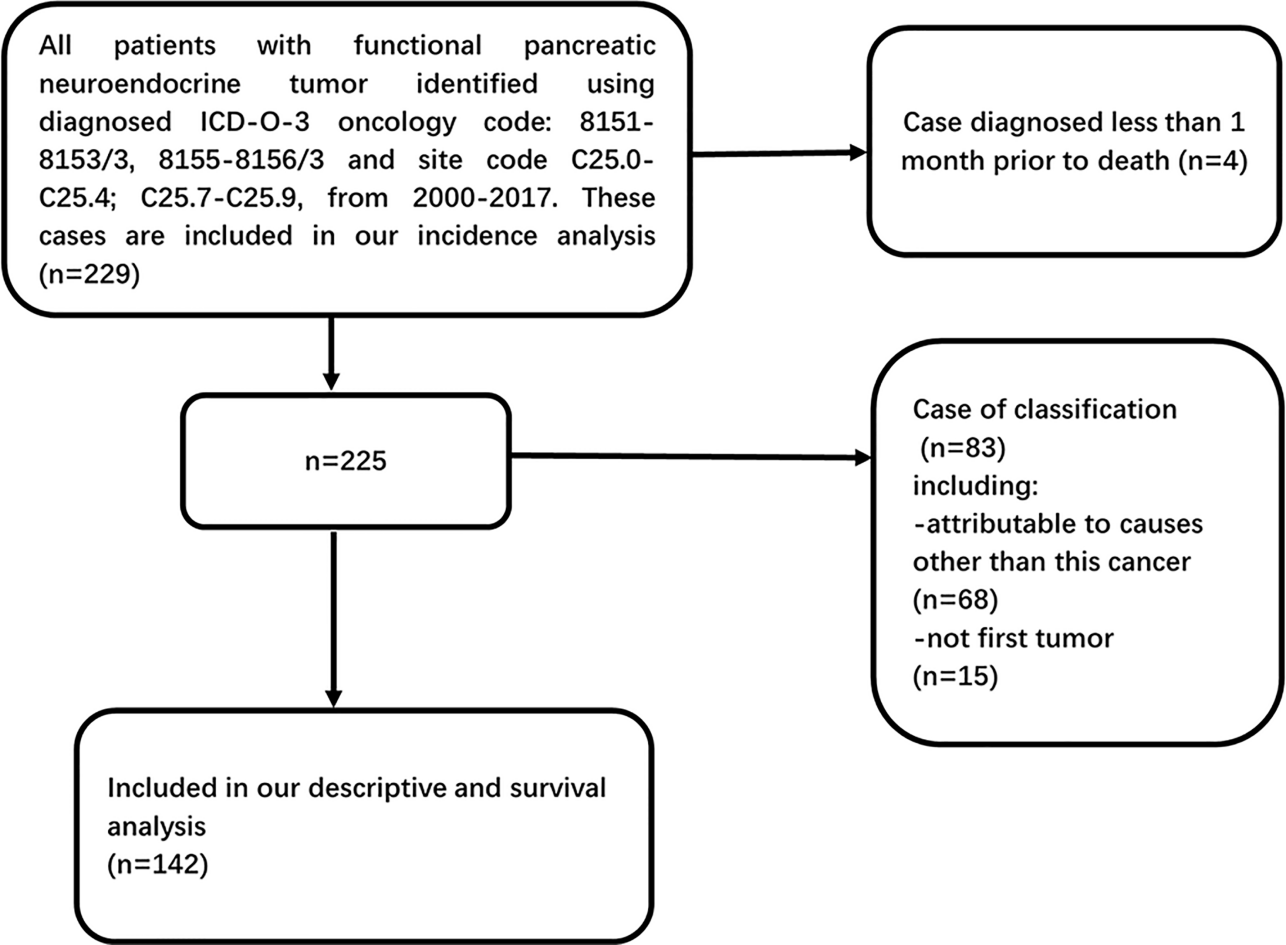
Flow diagram of inclusion and exclusion criteria in our descriptive (excluding incidence trends) and survival analysis within SEER database 2000–2017.

Data were obtained from the SEER program, a US cancer database that covers approximately 28% of the US population. The database records the incidence of malignant tumors and survival data in different areas of the US (9, 10). We used data from 2000 to 2017 in the SEER 18 registry to determine the incidence of F-PNETs and the trends, as well as for descriptive analyses. All cases of F-PNETs were given ICD-O-3 codes (8151/3, 8152/3, 8153/3, 8155/3, or 8156/3) and site codes (C25. 0–C25. 4; C25. 7–C25. 9). In survival analysis, patients were excluded if they had another primary tumor to avoid the misdiagnosis of F-PNETs due to metastasis; patients who were diagnosed within a month before death (diagnosis reported on the death certificate or diagnosed at autopsy) were included in the analysis of incidence trends, but they were excluded from the analyses that assessed survival because the survival time was recorded in months, not days; patients were also excluded who died from causes other than F-PNETs (**Figure 1**). We used the SEER staging classification, which provides consistent definitions over time, instead of the American Joint Committee on Cancer staging, which can change. The relevant SEER staging classifications are as follows: localized (cancer confined to the primary site), regionalized (cancer has spread to regional lymph nodes), and distant (cancer has spread to distant tissues and organs).

### Statistical analyses

We used SEER*Stat (version 8. 3. 9), a statistical software program, to determine the incidence of F-PNETs, the incidence-based (IB) mortality, and the patient characteristics, with 95% confidence intervals (CI). The incidence and IB mortality rates were age-adjusted using the US year 2000 population as the standard. The IB mortality rates were calculated, as opposed to the overall mortality rates, in order to reflect patients who had been diagnosed with F-PNETs prior to death, rather than at autopsy. This meant that in a given year, the F-PNETs IB mortality was just a proportion of the total number of deaths caused by F-PNETs.

We used the Joinpoint Regression Program (version 4. 7. 0; National Cancer Institute), which utilizes permutation analyses, to determine the APC and the average APC. This involved fitting joined straight lines (on a logarithmic scale) to the observed rates, to show trends in the incidence and IB mortality rates for different sexes, tumor sites, SEER stages, and histopathological types.

The SEER stage of the F-PNETs at diagnosis was missing for 5. 7% (n = 13) of the patients. Other variables were also missing for certain patients. We used multiple imputation by chained equations to fill in the missing values. The multiple imputation was primarily conducted to provide data for the tumor stage at diagnosis (SEER stage: 5. 7% missing), but the procedure also included other variables. These were the age at diagnosis, racial group, calendar year at diagnosis, surgery (1. 3% missing), tumor grade (57. 6% missing), survival time, vital status, tumor size, sex, tumor site, and the ICD-O-3 code (8151/3: insulinoma, malignant/8152/3: glucagonoma, malignant/8153/3: gastrinoma, malignant/8155/3: VIPoma, malignant/8156/3: somatostatinoma, malignant). It was assumed that data were missing at random (MAR); in other words, the probability of missingness depends on the observed data, not on the unobserved data. Although MAR cannot be verified statistically, it is a reasonable assumption (11), particularly in the context of this study. The procedure involved 100 imputations with 50 iterations for the burn-in period, which was shown to be sufficient using trace plots. For the survival analyses, cases with substandard data were removed, resulting in a reduction in the proportion of missing data. We therefore ran 40 imputations with 20 iterations for the burn-in period.

Age-adjusted F-PNETs incidence rates were calculated for each imputed dataset and combined using Rubin’s rules (12). For comparison, we also calculated age-adjusted F-PNETs incidence rates without multiple imputation by excluding cases with missing data for the tumor stage.

Demographic and clinical characteristics were summarized using descriptive statistics. Chi square tests were run to compare the categorical variables. Survival curves were estimated using the Kaplan–Meier method and compared using the log rank test. In addition, we used the Cox proportional hazards model to examine factors associated with mortality; this was run twice using the data with or without multiple imputation. All of the P values were two-tailed, and values less than 0. 05 were considered to be statistically significant. All of the statistical analyses were performed using SPSS (version 26. 0. 0).

## Results

### Description of patients and tumors

A total of 142 patients with F-PNETs met the study inclusion criteria (**Figure 1**). The demographic and tumor characteristics of the study population are shown in **Table 1**. The median age at diagnosis was 53. 5 years, and there were similar numbers of men (n = 74, 52. 1%) and women (n = 68, 47. 9%). Most of the patients were White (n = 111, 78. 2%), and this proportion remained relatively stable over the study period (from n = 32, 74. 4% in 2000-2005 to n = 40, 83. 3% in 2012-2017; P > 0. 05). In addition, there was a higher incidence of F-PNETs in White people than in the other ethnicity. The majority of the patients had localized disease (n = 58, 40. 8%), followed by distant disease (n = 41, 28. 9%) and regionalized disease (n = 36, 25. 4%). The proportion of patients with distant disease decreased significantly over the study period (from 44. 2% in 2000-2005 to 20. 8% in 2012-2017, P < 0. 05), while there was a marked increase in the proportion of patients with localized disease (from 23. 3% in 2000-2005 to 58. 3% in 2012-2017, P = 0. 001); the proportion of patients with regionalized disease remained relatively constant. Most of the patients had well-differentiated tumors (n = 53, 37. 3%), but the tumor grade was unknown for half of the patients (n = 71, 50. 0%). The proportion of well-differentiated tumors was found to increase over the study period (from 23. 3% in 2000-2005 to 54. 2% in 2012-2017, P < 0. 01), but there were no significant changes in the proportion of moderately differentiated, poorly differentiated, or undifferentiated tumors. It is important to note that differentiation in SEER is different from what is considered standard (the WHO classification). SEER did not provide information on mitotic count and Ki-67 index. The median size of the tumors was 2. 0 cm. Data for the tumor grade, size, and stage at diagnosis were missing for 50. 0%, 19. 7%, and 4. 9% of the patients, respectively.

**Table 1:** Demographic and pathological characteristics of the study population (2000–2017).

Variable	Total	2000-2005	2006-2011	2012-2017
Number of patients	142	43	51	48
Median age of diagnosis	53. 5	48	55	54. 5
Gender				
Female	68(47. 9)	20(46. 5)	27(52. 9)	21(43. 8)
male	74(52. 1)	23(53. 5)	24(47. 1)	27(56. 3)
Ethnicity				
White	111(78. 2)	32(74. 4)	39(76. 5)	40(83. 3)
Black	14(9. 9)	5(11. 6)	5(9. 8)	4(8. 3)
Other^a^	16(11. 3)	6(14. 0)	7(13. 7)	3(6. 3)
Unknown	1(0. 7)			1(2. 1)
SEER historic stage				
Localized	58(40. 8)	10(23. 3)	20(39. 2)	28(58. 3)
Regional	36(25. 4)	9(20. 9)	18(35. 3)	9(18. 8)
Distant	41(28. 9)	19(44. 2)	12(23. 5)	10(20. 8)
Unknown and blank	7(4. 9)	5(11. 6)	1(2. 0)	1(2. 1)
Grade				
Well differentiated	53(37. 3)	10(23. 3)	17(33. 3)	26(54. 2)
Moderately differentiated	16(11. 3)	3(7. 0)	3(5. 9)	10(20. 8)
Poorly differentiated	1(0. 7)	0(0. 0)	1(2. 0)	0(0. 0)
Undifferentiated	1(0. 7)	1(2. 3)	0(0. 0)	0(0. 0)
Unknown	71(50. 0)	29(67. 4)	30(58. 8)	12(25. 0)

a Other ethnicity include: American Indian/Alaskan Native, Asian/Pacific Islander.

### Overall incidence and mortality trends

**Figure 2:**
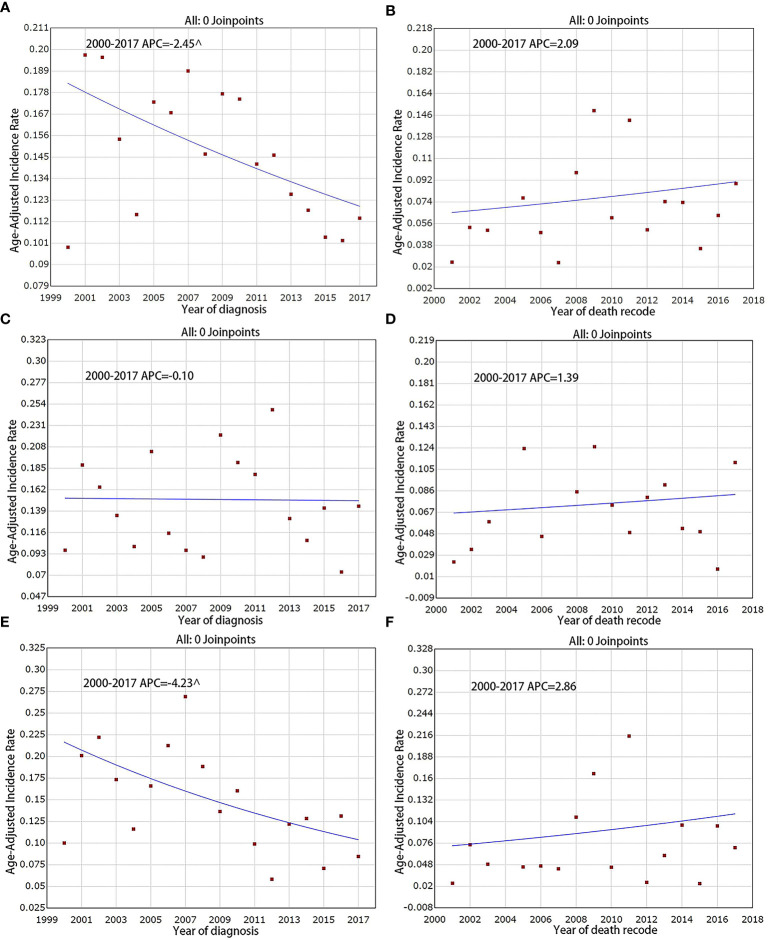
Incidence and IB mortality trends of F-PNETs overall and by sex, 2000–2017. **(A)** Incidence trends of F-PNETs. **(B)** IB mortality trends of F-PNETs.**(C)** Incidence trends of Male. **(D)** IB mortality trends of Male. **(E)** Incidence trends of Female. **(F)** IB mortality trends of Female. ^ mean that P < 0. 05.

The incidence of F-PNETs was found to decrease continually over the study period, from 2000 to 2017 (**Figure 2A**), with a final incidence of 0. 114 cases per million individuals. The APC (which reflects the slope or magnitude of the decrease) over this period was -2. 5% (95% CI [-4. 3, -0. 5], P < 0. 05). The IB mortality rate was seen to increase over the study period (**Figure 2B**), with an APC of 2. 1%, but this was not statistically significant (95% CI [-4. 0, 8. 6], P = 0. 50).

### Incidence trends according to sex

The overall incidence of F-PNETs was found to be lower in women than in men, with a declining incidence for women over the study period. In 2000, the incidence in men and women was 0. 097 and 0. 100 per million, respectively, compared with 0. 144 and 0. 085 per million in 2017. The decrease seen for the women was statistically significant, with an APC of -4. 2% (95% CI [-7. 4, -0. 9], P < 0. 05; **Figure 2E**); there was no significant decrease in the incidence for men (**Figure 2C**), with an APC of -0. 1% (95% CI [-3. 5, 3. 5], P = 1. 00). There were no significant changes in the IB mortality for either men or women over the study period; the IB mortality APC was 1. 4% (95% CI [-4. 8, 8. 0], P = 0. 60) for men and 2. 9% (95% CI: [-5. 3, 11. 7], P = 0. 50) for women (**Figures 2D, F**).

### Incidence trends according to the SEER stage

**Figure 3:**
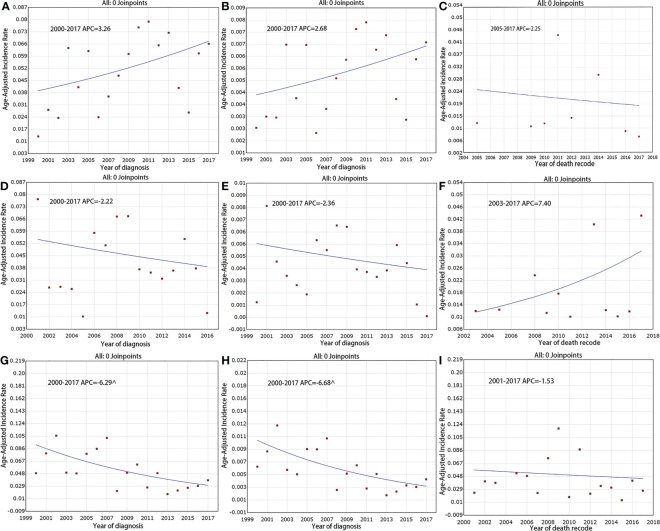
Incidence and IB mortality trends of F-PNETs by stage overall 2000–2017. **(A)** Incidence trends of localized disease (Before Imputation). **(B)** Incidence trends of localized disease (After Imputation). **(C)** IB mortality trends of localized disease. **(D)** Incidence trends of regional disease (Before Imputation). **(E)** Incidence trends of regional disease (After Imputation). **(F)** IB mortality trends of regional disease. **(G)** Incidence trends of distant disease (Before Imputation). **(H)** Incidence trends of distant disease (After Imputation). **(I)** IB mortality trends of distant disease. ^mean that P < 0. 05.

We assessed the incidence trends for the different SEER stages (localized/regionalized/distant). Overall, distant disease had the highest incidence and regionalized disease had the lowest incidence. Analyses were first carried out for the data prior to multiple imputation. It was found that the incidence of distant disease decreased significantly over the study period, with an APC of -6. 3% (95% CI [-10. 2, -2. 2], P < 0. 05) and a final incidence of 0. 038 per million (**Figure 3G**). The incidence of localized and regionalized disease did not change significantly, with APCs of 3. 3% (95% CI [-0. 7, 7. 4], P = 0. 10) and -2. 2% (95% CI [-7. 6, 3. 4], P = 0. 40), respectively (**Figures 3A, D**). For IB mortality, the APC was -2. 3% (95% CI [-21. 1, 21. 0], P = 0. 80) for localized disease, 7. 4% (95% CI [-2. 2, 17. 9], P = 0. 10) for regionalized disease, and -1. 5% (95% CI [-8. 8, 6. 4], P = 0. 70) for distant disease over the study period (**Figures 3C, F, I**).

The analyses were run again for the data with multiple imputation. The results showed that the downward trend in the incidence of distant disease became steeper, with an APC of -6. 7% (95% CI [-10. 4, -2. 8], P < 0. 05). For localized disease, the APC was 2. 7% (95% CI [-0. 8, 6. 2], P = 0. 1) and for regionalized disease, the APC was -2. 4% (95% CI [-7. 5, 3. 0], P = 0. 4; **Figures 3B, E, H**).

### Incidence trends according to the F-PNET subtype

**Figure 4:**
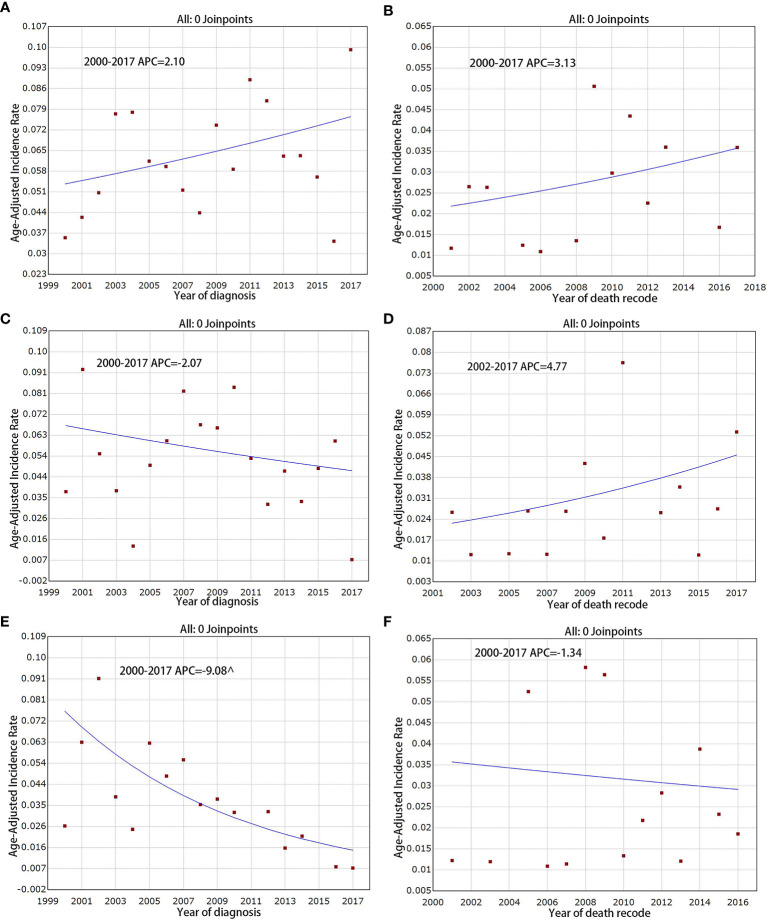
Incidence and IB mortality trends in Insulinoma, Gastrinoma and RF-PNETs overall 2000–2017. **(A)** Incidence trends in Insulinoma. **(B)** IB mortality trends in Insulinoma. **(C)** Incidence trends in Gastrinoma. **(D)** IB mortality trends in Gastrinoma. **(E)** Incidence trends in RF-PNETs. **(F)** IB mortality trends in RF-PNETs. ^ mean that P < 0. 05.

F-PNETs have several different subtypes. The incidence of the RF-PNETs (glucagonomas, VIPomas, and somatostatinomas) was found to decrease from 0. 026 per million in 2000 to 0. 007 per million in 2017 (**Figure 4E**), with an APC of -9. 1% (95% CI [-13. 5, -4. 4], P < 0. 05). However, the incidence of insulinomas and gastrinomas remained stable, with APCs of 2. 1% (95% CI [-0. 6, 4. 9], P = 0. 10) and -2. 1% (95% CI [-6. 3, 2. 4], P = 0. 30), respectively (**Figures 4A, C**). For IB mortality, there were slight, non-significant increases for both insulinomas and gastrinomas, with APCs of 3. 1% (95% CI [-2. 9, 9. 5], P = 0. 30) and 4. 8% (95% CI [-3. 0, 13. 2], P = 0. 20), respectively (**Figures 4B, D**); the APC for the RF-PNETs was -1. 3% (95% CI [-10. 4, 8. 7], P = 0. 80; **Figure 4F**).

### Incidence trends according to surgical treatment

A total of 100 of the 142 patients (70. 4%) underwent surgical resection. This included 86. 2% of the patients with localized disease, 86. 1% of those with regionalized disease, and 46. 3% of those with distant disease. The proportion of patients undergoing surgery did not change significantly between 2000–2005 and 2012–2017 (74. 4% vs 70. 8%, P > 0. 05).

### Long-term survival outcomes

Overall, the average survival time of patients with F-PNETs was 178 months (95% CI (163, 193)), and the 1-, 5-, and 10-year survival rates were 93. 6%, 80. 9%, and 74. 1%, respectively. The average survival time was seen to increase over the study period (**Supplementary Figure 1A**). Patients with well-differentiated or moderately differentiated tumors were found to have significantly longer survival times than patients with poorly differentiated or undifferentiated tumors (**Supplementary Figure 1B**); in addition, those with well-differentiated tumors survived longer than those with moderately differentiated tumors. Patients who underwent surgery were found to have a longer median survival time than those who without surgical treatment (P < 0. 01; **Supplementary Figure 1E**). In addition, the median survival time was significantly longer for patients with tumors no larger than 2 cm (**Supplementary Figure 1C**). Analyses were also conducted for the different SEER stages (**Supplementary Figure 1F**). It was found that patients with localized disease had the best prognosis and median survival times, while patients with distant disease had shorter survival times than those with regionalized disease (P < 0. 05). Analyses for the different F-PNET subtypes showed that survival times were significantly longer for insulinomas than for RF-PNETs (**Supplementary Figure 1D**).

Cox regression analysis using the data prior to multiple imputation (**Supplementary Table 1**) revealed that the tumor grade (poorly differentiated and undifferentiated), tumor size (> 2 cm), SEER stage (regionalized and distant), tumor type (gastrinomas and RF-PNETs), and surgery were all independently associated with F-PNETs mortality. However, when using the data following multiple imputation (**Table 2**), the tumor grade was no longer found to be independently associated with mortality.

**Table 2:** Univariate Cox’s proportional hazards model assessing factors associated with mortality after diagnosis of F-PNET with multiple imputation.

Risk Factors	Hazard ratios (HR)^a^	95% CI		P value
		Lower	Upper	
Stage				
Localized	Referent			
Regional	9. 08	1. 09	75. 58	0. 041
Distant	43. 84	5. 88	326. 92	<0. 001
Tumor size, cm				
< =2 cm	Referent			
> 2	2. 81	1. 05	7. 53	0. 040
Treatment				
No surgery procedure	Referent			
Resection	0. 36	0. 17	0. 74	0. 005
Pathological Types				
Insulinoma	Referent			
gastrinoma	2. 86	1. 15	7. 08	0. 023
Other ^b^	3. 55	1. 32	9. 54	0. 012

a HRs greater than 1. 0 indicate a higher risk of death.

b Other Pathological Types include: Glucagonoma, Vipoma, Somatostatinoma.

## Discussion

The relative frequency of the different PNETs appears to be changing in recent reports. However, changes in the incidence and mortality rates of F-PNETs over time have remained unclear. Here, we conducted the largest population-based epidemiological study of F-PNETs to date, and we revealed the incidence and mortality trends for the first time. The results are of relevance to therapeutic approaches as well as preventive strategies.

Our analyses showed that the incidence of F-PNETs decreased steadily over the study period, from 2000 to 2017. However, as far as we know, there is no clear strategy to prevent F-PNETs. The prevention of genetic diseases may affect the incidence of F-PNET. Four different inherited syndromes are associated with the development F-PNETs; patients with Multiple Endocrine Neoplasia type 1(MEN1); von Hippel Lindau Disease (VHL); von Recklinghausen disease (VRH) (neurofibromatosis 1) (NF-1) and patients with tuberous sclerosis (13). In addition, recent data show that the widespread use of proton pump inhibitors (PPIs) is making the diagnosis of gastrinomas more difficult (14, 15). This result of F-PNET contrasts strikingly with the incidence trends found for NF-PNETs (8, 16), where the incidence has been found to be increasing rapidly (8). This difference may relate to the pathophysiological characteristics of the different tumors. Our study also analyzed the IB mortality for F-PNETs and found that it remained at a low level over the study period, with a slight but non-significant increase. This contrasts with our previous study on NF-PNETs, where the IB mortality showed a sustained increase (8). These differences are consistent with previous studies that have shown a more favorable prognosis for F-PNETs than for NF-PNETs, with better treatment outcomes (17).

Our analyses examined the incidence of F-PNETs over time for the different SEER stages. We found that the incidence of distant disease decreased rapidly, having the highest incidence at the beginning of the study period, but ending with an incidence lower than for localized disease. This decrease may result from recent improvements in tumor localization, which enable tumors to be detected early. The techniques that can be used include somatostatin receptor scintigraphy (SRS), computed tomography scans, magnetic resonance imaging, selective angiography, and endoscopic ultrasound (18). In line with previous studies (4), we found that insulinomas and gastrinomas are the most common F-PNETs. The incidence of these remained stable over the study period, but the incidence of RF-PNETs (glucagonomas, VIPomas, somatostatinomas) decreased significantly. Insulinomas and gastrinomas are therefore the tumors that require the most attention because they continue to affect more people than other F-PNETs. We examined the survival times for the different F-PNET subtypes, and found that the prognoses for patients with RF-PNETs or gastrinomas were significantly worse than for patients with insulinomas. This may be because most RF-PNETs are malignant and liver metastases have been reported to be common (19, 20); pancreatic gastrinomas show local invasion and/or proximal lymph node metastases, as well as frequent liver metastases (22%–35%) (21).

Our results showed that the incidence of F-PNETs was similar for men and women, but there was a significant decrease in the incidence for women over the study period. There may be significant gender differences in the incidence of F-PNETs over time. A previous US study reported a higher incidence of PNETs in men (22). Our study also found that there was a higher incidence of F-PNETs in White people than in Black people. This contrasts with our previous study on NF-PNETs (8), which showed a higher incidence in Black people. When screening for pancreatic malignancy, we might therefore be more vigilant for F-PNETs in White people, and NF-PNETs in Black people.

In our study, a tumor larger than 2 cm was independently associated with an increased risk of death. In line with this, previous studies have shown that insulinomas of at least 2 cm predict metastatic disease (23), and gastrinomas larger than 3 cm indicate a poor prognosis (24). These findings indicate that a large tumor size (> 2 cm) affects the biological behavior of F-PNETs resulting in poorer outcomes; conversely, smaller tumors are associated with more favorable outcomes.

We found that the average survival time following the diagnosis of F-PNETs increased over the study period. This indicates that recent advances in surgical treatment, interventional radiology, ablation techniques (25), and targeted therapies (26, 27) have improved the treatment and hence the prognosis of F-PNETs. We identified various factors that are independently associated with an increased risk of death: a large tumor size (> 2 cm), regionalized or distant disease, and gastrinomas or RF-PNETs. These factors could be used to inform clinical decision-making and risk assessment. We also found that surgery increased the survival times of patients with F-PNETs, in line with previous studies showing that surgery can increase disease-related survival (28–30). Previous studies have described the problems that can arise when cases with missing data are excluded, such as potential bias (31, 32). In our study, we used multiple imputation to deal with the missing data, thus enabling the use of the full dataset (33). The results of the statistical analyses should therefore have been more accurate, with less interference from errors and missing data. We also analyzed trends by combining multiple imputation with age adjustment, which should have led to more accurate results.

The main strength of this study lies in it being a large population-based study that assesses trends in the incidence and prognosis of F-PNETs for the first time. In addition, the use of multiple imputation leads to more accurate results. The limitations include the fact that the SEER database does not provide information concerning the immunohistochemistry results, disease complications, comorbidities, and other clinical details, such as the occurrence of biliary obstruction.

## Conclusions

This study showed that the incidence of F-PNETs has decreased in recent years. The IB mortality has remained at a low level, showing that the treatments are effective. Several risk factors were identified that are independently associated with mortality. These indicate that early diagnosis and surgical intervention are paramount for a favorable prognosis.

## Data availability statement

Publicly available datasets were analyzed in this study. This data can be found here: https://seer.cancer.gov/.

## Author contributions

Research design and project supervision: WL and YL; Data collection, statistical analysis and writing: SL, JW, JG, and ML; Quality control of data and algorithms: SL, CW, JY, CS, and JL; Data analysis and interpretation: JW and EL; Literature review and manuscript proofreading: SL, LW, LZ, and FZ. All authors contributed to the article and approved the submitted version.
