# Supplementary material for: Epidemiological trends for functional pancreatic neuroendocrine tumors: A study combining multiple imputation with age adjustment
Source: Front Endocrinol (Lausanne). 2023 Apr 11;14:1123642. doi: 10.3389/fendo.2023.1123642 (PMC10126336; doi:10.3389/fendo.2023.1123642)
Supplement: Supplementary file 1 [file DataSheet_1.docx]

Supplementary Material

Epidemiological Trends for Functional Pancreatic Neuroendocrine Tumors: A Study Combining Multiple Imputation with Age Adjustment

**Shuaiwu Luo^1†^ (M Med), Jiakun Wang ^1†^ (M Med), Linquan Wu^2†^ (M Med), Cong Wang^1^(M Med), Jun Yang^1^(M Med), Min Li^1^ (M Med), Jin Ge^2^ (M.D.), Chi Sun^2^(M.S.N.), Enliang Li^2^(M.D.), Jun Lei^2^(M.D.), Wenjun Liao^2^*(M.D.)**

*Corresponding author:

Wenjun Liao; Tel: 86-13870633069; e-mail: [liaowenjun120@163.com](mailto:liaowenjun120@163.com)

## 1. Supplementary Table

**Supplementary Table 1****:** Univariate Cox’s proportional hazards model assessing factors associated with mortality after diagnosis of F-PNET without multiple imputation

| Risk Factors | Hazard ratios (HR)^a^ | 95%CI | | P value |
| --- | --- | --- | --- | --- |
|  |  | Lower | Upper |  |
| Stage |  |  |  |  |
| Localized | Referent |  |  |  |
| Regional | 9.00 | 1.08 | 74.74 | 0.042 |
| Distant | 44.81 | 6.04 | 332.30 | <0.001 |
| Tumor size, cm |  |  |  |  |
| ≤ 2 cm | Referent |  |  |  |
| > 2 | 3.69 | 1.47 | 9.24 | 0.005 |
| Treatment |  |  |  |  |
| No surgery procedure | Referent |  |  |  |
| Resection | 0.36 | 0.17 | 0.74 | 0.005 |
| Pathological Types |  |  |  |  |
| Insulinoma | Referent |  |  |  |
| gastrinoma | 2.86 | 1.15 | 7.08 | 0.023 |
| Other ^b^ | 3.55 | 1.32 | 9.54 | 0.012 |
| Grade |  |  |  |  |
| Well differentiated, I | Referent |  |  |  |
| Moderately differentiated, II | 5.00 | 0.69 | 35.99 | 0.110 |
| Poorly differentiated, III | 129.22 | 6.39 | 2612.2 | 0.002 |
| Undifferentiated, IV | 259.68 | 259.68 | 7105.19 | 0.001 |

^a^ HRs greater than 1.0 indicate a higher risk of death

^b^ Other Pathological Types include: Glucagonoma, Vipoma, Somatostatinoma

## 2. Supplementary Figure

**
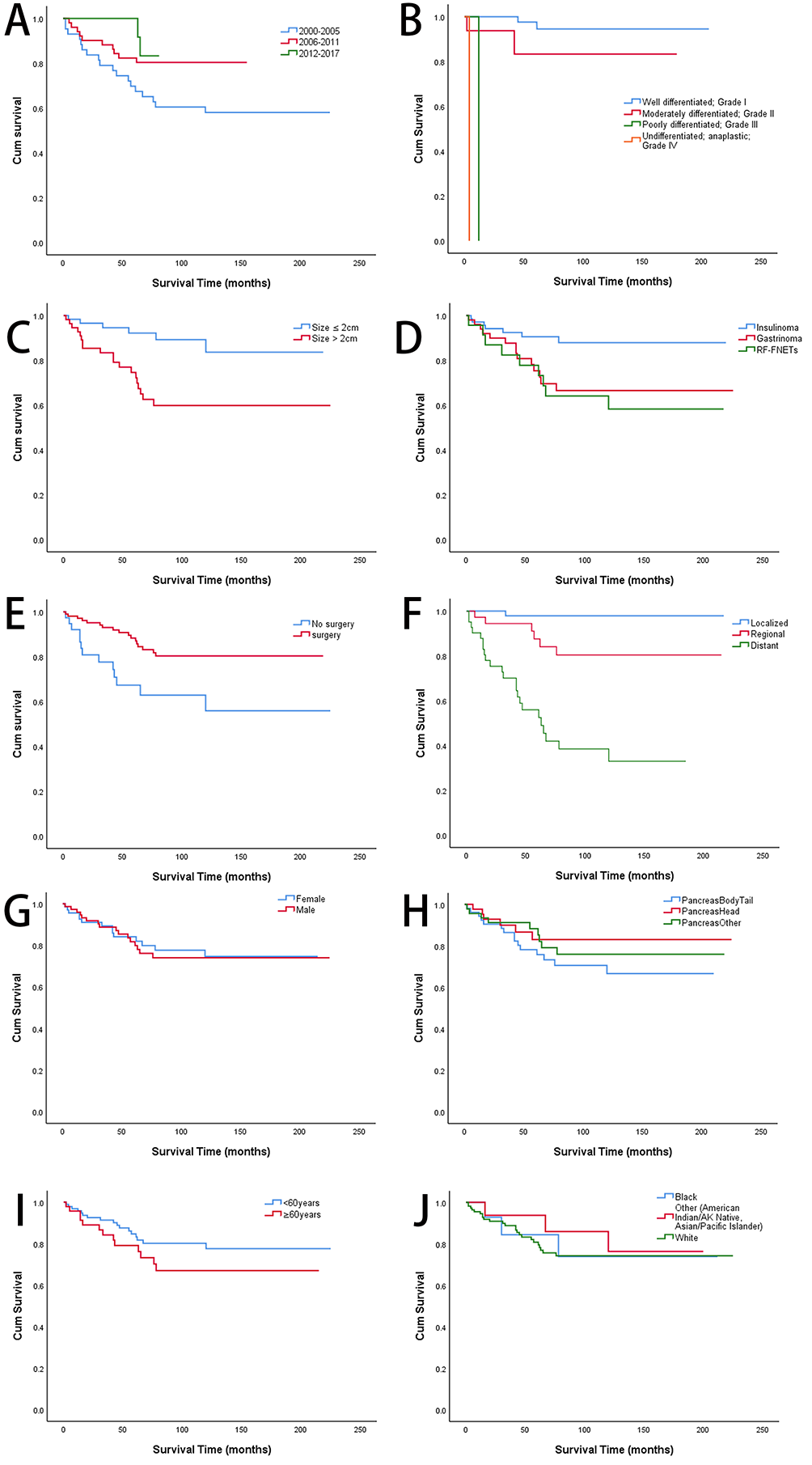
**

**Supplementary Figure 1.** Long-term survival outcomes using Kaplan–Meier’s analysis: **A** Survival time in study duration, Graph shows the median survival duration increased with time (P < 0.05). **B** Long-term survival outcomes in Well differentiated, Moderately differentiated, Poorly differentiated and Undifferentiated F-PNETs. The survival was better in Well differentiated and Moderately differentiated (p < 0.01) compared with Poorly differentiated and Undifferentiated F-PNETs. **C** Long-Term Survival Outcomes in tumor size of F-PNETs (p < 0.01). **D** long-term survival outcomes in Insulinoma, Gastrinoma and RF-PNETs. The survival was better in Insulinoma (p < 0.05) compared with Gastrinoma and RF-PNETs. **E** Long-term survival outcomes in treatment of F-PNETs (p < 0.01). **F** Long-term survival outcomes in localized, regional and distant F-PNETs. Graph shows decreasing survival from localized to distant (p < 0.05). The P values reported for survival analysis refers to comparison among all stage. **G** Long-Term Survival Outcomes in male and female of F-PNETs (p = 0.86). **H** Long-term survival outcomes in tumor location of F-PNETs (P > 0.10). **i** Long-term survival outcomes in age<60 years and age ≥60 years of F-PNETs (p = 0.18). **j** Long-term survival outcomes in race of F-PNETs (P > 0.10).
